# Biomechanical and Metabolic Effectiveness of an Industrial Exoskeleton for Overhead Work

**DOI:** 10.3390/ijerph16234792

**Published:** 2019-11-29

**Authors:** Thomas Schmalz, Jasmin Schändlinger, Marvin Schuler, Jonas Bornmann, Benjamin Schirrmeister, Andreas Kannenberg, Michael Ernst

**Affiliations:** 1 Clinical Research & Services/Biomechanics, Otto Bock SE & Co. KGaA, 37075 Göttingen, Germany; Michael.Ernst@ottobock.com; 2 Private University of Applied Sciences, 37075 Göttingen, Germany; jasmin.schaendlinger1@pfh.de (J.S.); mschuler@pfh.de (M.S.); 3 Global Research, Otto Bock SE & Co. KGaA, 37115 Duderstadt, Germany; Jonas.Bornmann@ottobock.de (J.B.); Benjamin.Schirrmeister@ottobock.de (B.S.); 4 Clinical Research & Services, Otto Bock Healthcare LP, Austin, TX 78758, USA; Andreas.Kannenberg@ottobock.com

**Keywords:** exoskeleton, assistive device, EMG, shoulder, Ergonomics, Biomechanics, work-related musculoskeletal disorders, Occupational health

## Abstract

Overhead work activities can lead to shoulder pain and serious musculoskeletal disorders (WMSD), such as rotator cuff injury and degeneration. Recently developed exoskeletons show promising results in supporting workers in such activities. In this study, a novel exoskeleton was investigated for two different overhead tasks with twelve participants. To investigate the effects of the device, electromyographic (EMG) signals of different shoulder and adjacent muscles as well as kinematic and metabolic parameters were analyzed with and without the exoskeleton. The mean EMG amplitude of all evaluated muscles was significantly reduced when the exoskeleton was used for the overhead tasks. This was accompanied by a reduction in both heart rate and oxygen rate. The kinematic analysis revealed small changes in the joint positions during the tasks. This study demonstrated the biomechanical and metabolic benefits of an exoskeleton designed to support overhead work activities. The results suggest improved physiological conditions and an unloading effect on the shoulder joint and muscles which are promising indicators that the exoskeleton may be a good solution to reduce shoulder WMSD among workers who carry out overhead tasks on a regular basis.

## 1. Introduction

Work-related musculoskeletal disorders (WMSD) make up a large proportion of occupational diseases, especially in countries with a large industrial production sector [1]. In this context, repetitive overhead work has been clearly identified and documented as a type of work that presents a high risk of WMSD [2]. It was reported that 69% of patients with shoulder pain did jobs involving large anteversion angles in the shoulder joint [3], which led to the inference that such postures were directly related to a significant increase in stress to the joint [3,4]. Ergonomic arrangements, such as the use of handling tools (e.g., hand-held manipulators, industrial robots), proved to be advantageous in some areas. However, the major disadvantages of these approaches include low user acceptance due to time delays, increased “movement effort” as well as the lack of necessary flexibility while working [5]. This demonstrates that industrial robots are not fully able to adequately replace the necessary flexibility provided by human movements in production thus far.

Newly developed exoskeletons, which are also known as industrial exoskeletons, could represent beneficial alternatives due to their light weight, high flexibility and wearing comfort. These devices are external structures worn on the body that provide support for a range of tasks and may improve the user’s performance. They have to fulfill high demands on functionality, safety, comfort, and user acceptance in daily work and should therefore be individually adaptable to the anthropometry of the user. Such flexible, space-saving solutions usually do not require modifications to the workplace. Therefore, industrial exoskeletons are attracting great interest for various industrial applications [6]. Especially for overhead work activities, a number of systems is commercially available, e.g., EksoVestTM (Ekso Bionics, Richmond, CA, USA), AirframeTM (Levitate Technologies, San Diego, CA, USA), ShoulderX (SuitX, Emeryville, CA, USA), SkelEx (Skel-Ex, Rotterdam, Netherlands) and PAEXO (Ottobock, Duderstadt, Germany). These industrial exoskeletons are passive devices that utilize mechanical and/or pneumatic/hydraulic parts to support their users by transferring forces from the upper limbs to the pelvis and/or by storing and releasing energy during movements.

Several studies have investigated exoskeletons for overhead work and their effect on the physical stress to the shoulder joint and muscles, e.g., [7,8,9,10,11,12]. To evaluate these effects, mainly the activation (EMG) of the shoulder muscles was analyzed in these studies. A significant reduction in the EMG amplitude was reported and associated with a reduction in physical stress to the shoulder joint. Another parameter to investigate possible user benefits is the metabolic energy consumption in work-related activities. This rarely used method indicates the metabolic effort a worker has to put into the execution of such activity [12,13]. Despite positive reports describing the benefits exoskeletons offer during overhead work, evidence regarding their functionality as well as possible adverse effects have become the subject to scientific discussion [14,15,16,17]. For example, it is important that the load transfer and possible kinematic changes do not significantly increase strain to other parts of the body, e.g., the lower back or pelvis.

In this study, two typical overhead activities—screwing nuts and drilling—were investigated with and without using an exoskeleton. To test the hypothesis that the exoskeleton facilitates these tasks, electromyographic signals of ten muscles as well as general metabolic parameters were measured during the tasks. Furthermore, kinematic data of the upper body were captured to determine possible effects of the system on motion patterns.

## 2. Materials and Methods

### 2.1. Exoskeleton

**Figure 1:**
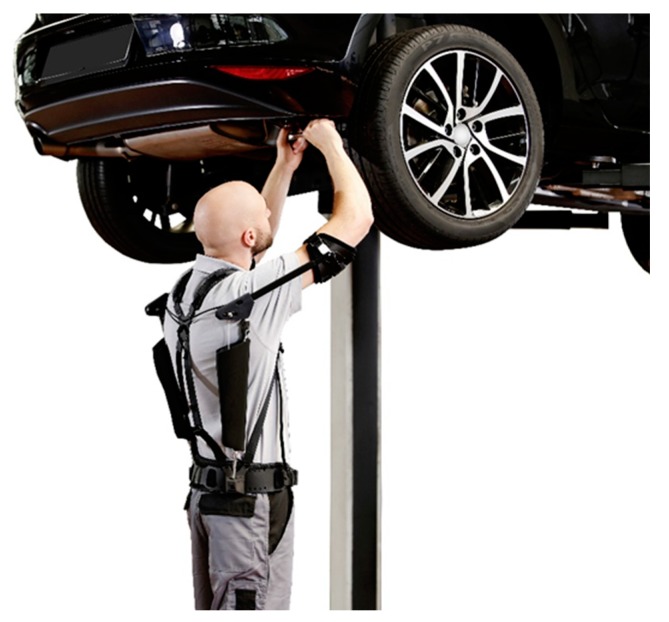
The passive exoskeleton Paexo used in industrial environment.

This study utilized the PAEXO passive exoskeleton (Ottobock SE & Co. KGaA, Duderstadt, Germany; weight: 1.9 kg), that has been designed to support overhead work activities [18]. The exoskeleton transfers a portion of the user’s arm weight to a hip belt while maintaining the user’s freedom of movement. The weight transfer is designed to compensate for the gravitational shoulder torque. The maximum level of support is thus provided at an elevation angle of around 90° (upper arm in horizontal position) and decreases to zero when the arm is lowered to the side of the body. The PAEXO consists of a support structure with an expander that generates the supportive torque via an adjustable lever arm. When the arm is lowered the expander is under maximum tension. In this position, the effective lever arm is zero, meaning that no supportive torque is generated. The PAEXO is connected to the upper arm by an arm brace and a hip belt (Figure 1). A stabilizing structure keeps the exoskeleton close to the body while allowing it to move in a manner comparable to that of the scapula. The movement of the trunk and upper extremities is largely unrestricted. The hip belt, arm brace, and support bar are adjustable for an optimal fit to the user. The level of support can be fine-tuned continuously to adapt to different arm weights or to compensate for the extra weight of tools. For the setting during the trials, the support force of the exoskeleton was adjusted to compensate for 70% of the individual user’s shoulder torque caused by the weight of the arm in the “calibration position” of 105° elevation. The weight of every user’s arm was estimated according to the individual body weight using anthropometric tables [19]. The procedure allowed for a comparison of the results as all users had the same level of relative support.

### 2.2. Participants

Twelve healthy subjects volunteered for the study (six male, six female, age: 24 ± 3y, height: 176 ± 15 cm, weight: 73 ± 15 kg). None of the participants was familiar with the use of exoskeletons. They provided written, informed consent before participating in the tests. The study was approved by the ethical committee of the medical faculty at the University of Göttingen (study number 23/6/18) and was conducted in accordance with the Declaration of Helsinki.

### 2.3. Simulation of Overhead Work Under Laboratory Conditions

**Figure 2:**
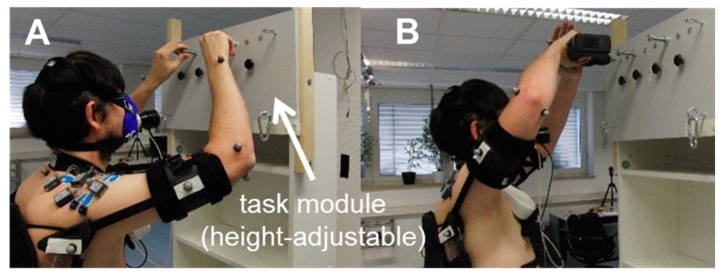
Performing the overhead work activities under lab conditions: (**A**) Screwing nuts T1 and (**B**) drilling T2.

In order to simulate overhead work activities that closely approximated industrial workflows, a modified rack with a height-adjustable task module was installed in the motion capture laboratory (Figure 2). The height of the module was individually adjusted at eye level, so all users had the same working conditions. The participants performed two tests:

**T1:** Screwing nuts continuously. Only negligible alterations in the shoulder and elbow joint angles were expected during this task, so it was categorized as a test under static conditions (SC, Figure 2A).

**T2:** Drilling using an electric drill (1.3 kg). Minor alterations in the shoulder and elbow joint angles were expected during this task, so it was categorized as a test under semi-static conditions (SSC, Figure 2B).

### 2.4. Test Design

**Figure 3:**
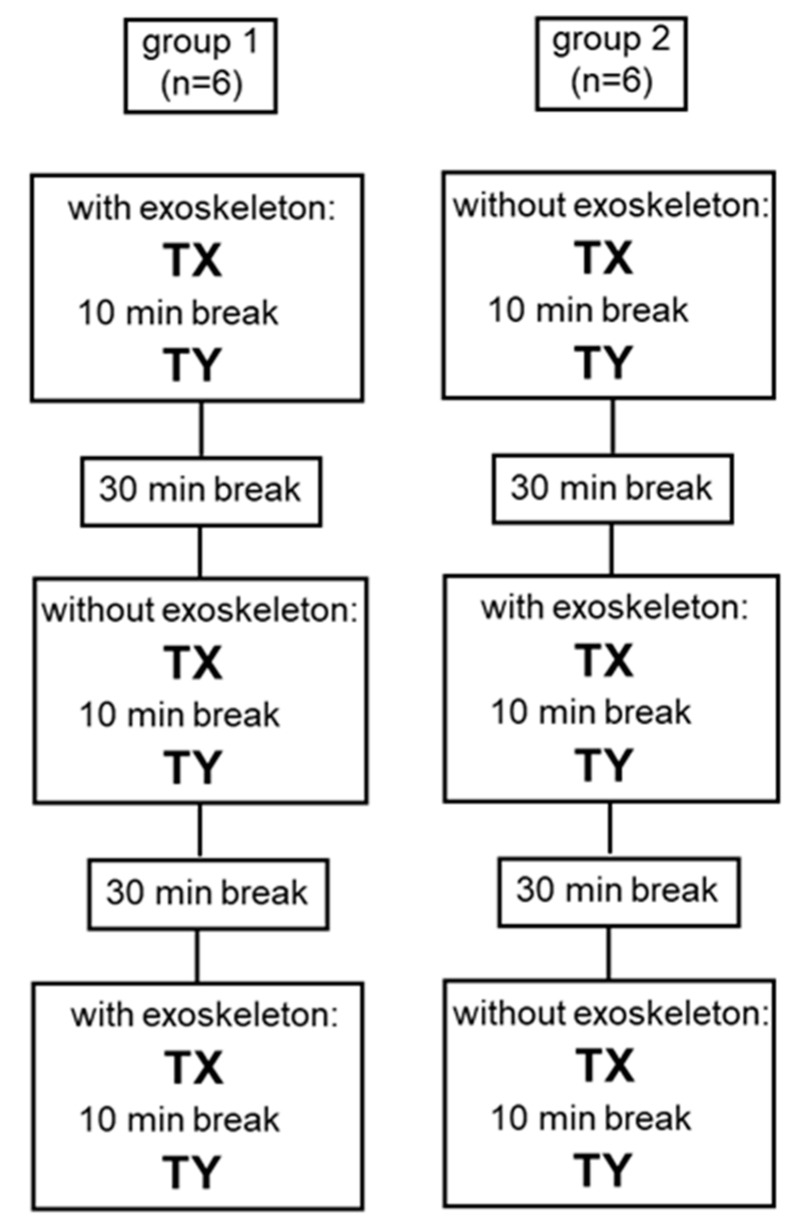
Sequence of the measurement session (individually randomized task order: x = 1 and y = 2 or x = 2 and y = 1, e.g., T1 followed by T2 for the entire session or T2 followed by T1 for the entire session). Subjects of group 1 started the session with the exoskeleton (WE), whereas subjects of group 2 started the session without the exoskeleton (WOE).

After being informed about the study design, the participants received instructions regarding the correct execution of T1 and T2. Subsequently, they completed a practical training exercise (duration: 20 min) while wearing the full set of measurement equipment. After a 30-min break, the study measurements commenced. The participants were randomly assigned to two groups to minimize the effects of adaptation (Figure 3): Group one started with using the exoskeleton (WE) for T1 and T2. After a 30-min break, the test was repeated without the device (WOE), followed by a second break. Finally, the initial test with the exoskeleton was repeated once more. Group two executed the whole assessment session in reversed order, i.e., these participants conducted the tests twice without and once with the exoskeleton. The order of tasks (T1 and T2) was also randomized for each participant for the first test and kept the same for the following tests (Figure 3). To guarantee that a physiologically steady state was achieved during T1 and T2, a duration of 5 min was chosen for both tasks [20]. Directly before each task (T1 and T2) of every block, the participants remained in a seated position in front of the rack for 2 min to determine their metabolic rate at rest.

### 2.5. Measurement Devices

#### 2.5.1. Metabolic Parameters

The MetaMax3b spiroergometry system (Cortex Biophysik, Leipzig, Germany) was used to measure the oxygen rate in a breath-by-breath mode. Data was recorded with a portable device equipped with all sensors (oxygen concentration, carbon dioxide concentration, breathing gas volume). Simultaneously, the heart rate was measured with the T31 sport tester (Polar Electro Oy, Kempele, Finland).

#### 2.5.2. Kinematics

**Figure 4:**
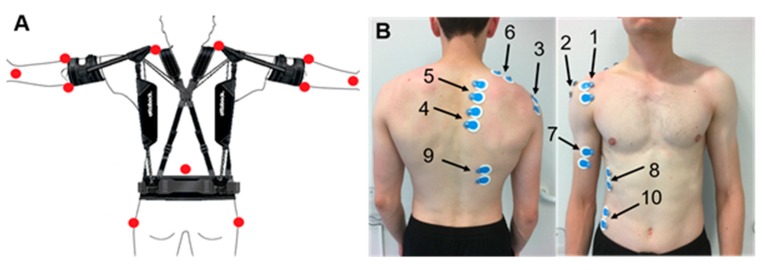
(**A**) Marker placement for the kinematic analysis of shoulder and elbow joints (see main text) and (**B**) EMG electrodes applied to: 1–3 deltoid (anterior, medius, posterior) muscle, 4–6 trapezius (ascendens, transversalis, descendens) muscle, 7 biceps brachii muscle, 8 serratus anterior muscle, 9 latissimus dorsi muscle, 10 obliquus externus abdominis muscle.

Kinematic data was recorded during the first and the last 30 s of T1 and T2 using an optoelectronic system (12 Bonita cams, VICON, Oxford, UK, measurement frequency 200 Hz). In order to reconstruct the joint angles, passive markers were placed on twelve anatomical landmarks: acromion (right/left), medial and lateral humeral epicondyle (right/left), styloid process of the ulna (right/left), C7, sacrum and major trochanter (right/left, Figure 4, left).

#### 2.5.3. Electromyography

The wireless Telemyo DTS system (NORAXON, Scottsdale, AZ, USA, measurement frequency 1000 Hz) was used to measure electromyographic signals throughout the entire duration of T1 and T2. Surface electrodes of the Blue Sensor P-00-S/50 (AMBU, Ballerup, Danmark) type were placed on the following muscles on the right side of the trunk according to the SENIAM convention [21,22] (Figure 4, right): anterior, medial, and posterior deltoid muscle, ascending, transversal, and descending trapezius muscle, biceps brachii muscle, anterior serratus muscle, latissimus dorsi and external obliquus abdominis muscle. Kinematic and electromyographic measurement devices were synchronized by a trigger.

### 2.6. Data Processing

#### 2.6.1. Metabolic Parameters

The values recorded in the last minute before starting T1 or T2 were averaged in order to determine the resting values for heart rate and oxygen rate. The values in the last minute of T1 and T2 were also averaged using the recorded data. Group means were calculated based on all the individual data.

#### 2.6.2. Kinematics

The 3D coordinates of the markers were used as input data for DYNAMICUS, a biomechanical full-body model consisting of 43 segments and 42 joints implemented in the multibody software package ALASKA (Institut für Mechatronik, Chemnitz, Germany).

The measured markers were linked with virtual markers placed on an individualized human body model. Based on this model, 3D joint angles were reconstructed by an inverse kinematic/dynamic algorithm.

In order to compare the test situations with (WE) and without (WOE) the exoskeleton, the mean values throughout all the analyzed periods were determined for the shoulder anteversion and abduction angles and the elbow flexion angle. The procedure was also executed separately for the first and the last 30-s period to obtain information on joint angle alterations over the 5-min trial in T1 and T2. Group means were determined using the individual parameters.

Shoulder anteversion and abduction angles and elbow flexion angles were estimated for a kinematic comparison of WE and WOE. First, the mean values of all analyzed periods were determined for T1 and T2. Second, the mean values of T1 and T2 were calculated separately for the first and the last 30-s period so the joint angle adaptations over the 5-min trials could be evaluated. Group means were then calculated for all values.

#### 2.6.3. Electromyography

The EMG raw data was rectified and ECG artefacts eliminated by specific algorithms [23]. The EMG signal was subsequently smoothened by an RMS filter (window size 100 ms), and amplitude means were calculated over the entire 5-min period of T1 and T2. Group means were once again determined.

Since T1 was categorised as a static task, it was possible to determine the Muscle Fatigue Index (MFI, [24]), a parameter for evaluating symptoms of fatigue over time in static tasks. The MFI describes a shift of the mean EMG frequency towards lower frequencies if a muscle fatigues over a longer period of work [24]. To determine the MFI parameter, T1 was divided into periods (P) of 10 s. The mean frequency was calculated for each P. The MFI is the gradient of the linear regression between mean frequency and time (measure [(sP)^−1^]. Negative MFI values indicate muscle fatigue.

### 2.7. Statistics

The non-parametric WILCOXON test was used for all analysed group means to identify significant differences between WE and WOE conditions.

## 3. Results

### 3.1. Metabolic Parameters

**Figure 5:**
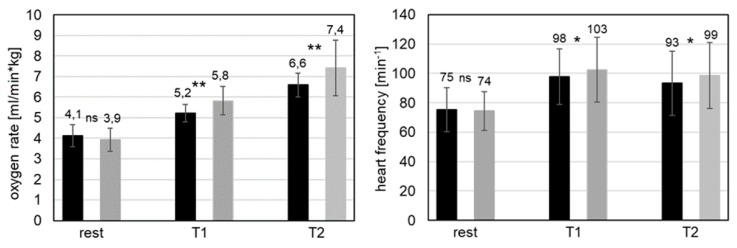
Mean metabolic parameters for T1, T2 and rest (measured in the last minute before starting T1 and T2); black: WE condition, grey: WOE condition; ns: no significant difference; *, **: significant difference with *p* < 0.05, *p* < 0.01).

No significant differences between WE and WOE conditions were found for both the heart and the oxygen rate at rest before starting the overhead work (resting metabolic rate; Figure 5). 

The metabolic steady state values showed significant differences between WE and WOE conditions for T1 and T2 with a similar magnitude. The heart rate was significantly decreased by 6% and 5%, respectively, when the exoskeleton was used *(p* < 0.05, Figure 5). The measured reduction in oxygen rate with the device was 11% and 12%, respectively (*p* < 0.01, Figure 5).

### 3.2. Kinematics

**Figure 6:**
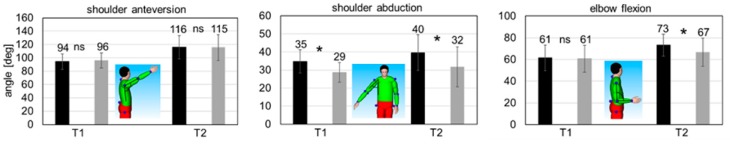
Mean joint angles for T1 and T2 (black: WE condition, grey: WOE condition; ns: no significant difference; *: significant difference with *p* < 0.05).

The mean shoulder anteversion angle was nearly the same for both conditions (WE and WOE) in both tasks (Figure 6). However, the shoulder joint was in approximately 20° more anteversion during the semi-static overhead work activity (compare T1 and T2 in Figure 6). With respect to the mean shoulder abduction angle, significantly increased mean values for WE were seen in both T1 and T2 (6° and 8°, *p* < 0.05). The abducted position was slightly larger during the semi-static overhead work activity (Figure 6). For the T1 static task, the mean elbow flexion angle was almost identical for WE and WOE conditions. During the T2 semi-static task, the mean elbow flexion angle was significantly increased for WE as compared to WOE (7°, *p* < 0.05, Figure 6).

Kinematic adaptations over time within the single trials (5-min trial duration) were not observed, with the exception of one parameter. In the case of T2, the shoulder anteversion angle significantly decreased by 7° in the final period, both for WE and WOE conditions (*p* < 0.05).

### 3.3. Electromyography

**Figure 7:**
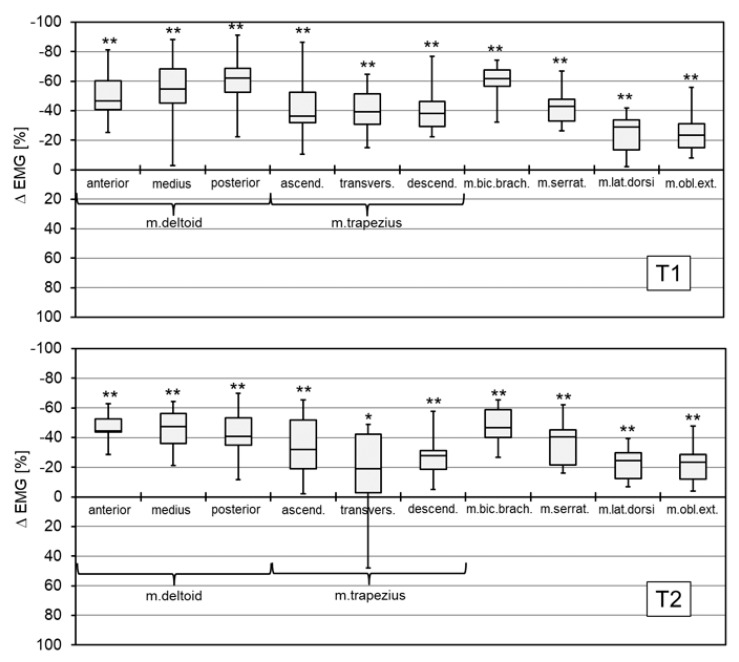
Box plots showing the difference of the mean EMG amplitude (ΔEMG = EMG (WE) − EMG (WOE)) for T1 (upper figure) and T2 (lower figure) over the entire 5 min test period (negative values: EMG amplitude for WE is reduced compared to WOE; *, **: significant difference between the mean WE and WOE amplitudes with *p* < 0.05, *p* < 0.01).

For both the T1 and T2 tasks, the mean EMG amplitude of all assessed muscles was significantly reduced when the exoskeleton was used (*p* < 0.01, Figure 7). The effects were more pronounced in the T1 static task with a mean amplitude reduction of between 61% and 22% (for details see Figure 7). For the T2 semi-static task, the observed reduction in mean amplitudes was between 48% and 22% (for details see Figure 7).

**Figure 8:**
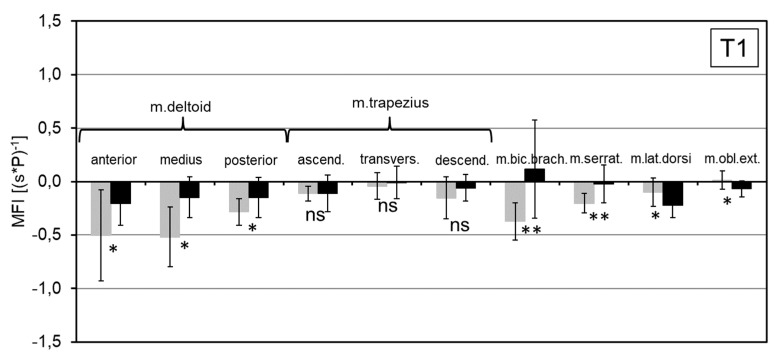
Mean MFI for T1 (black: WE condition, grey: WOE condition; ns: non significant difference; *, **: significant difference with *p* < 0.05, *p* < 0.01).

The muscle fatigue index (MFI) was significantly reduced by between 0.07 and 0.30 (s*P)^−1^ for the three parts of deltoid muscle, by 0.67 (s*P)^−1^ for the biceps brachii muscle and by 0.16 (s*P)^−1^ for the anterior serrator muscle (Figure 8). These changes indicate reduced local muscle fatigue when the exoskeleton was used (*p* < 0.05). No significant MFI changes were found for the parts of thrapezius muscle. The changes in MFI for the latissimus dorsi and obliquus muscles indicated a slight increase in local muscle fatigue for the WE condition (*p* < 0.05).

## 4. Discussion

This study investigated the biomechanical and metabolic effects of a passive exoskeleton under laboratory conditions for two specific tasks representing typical overhead work activities. The use of static and semi-static tasks facilitated the interpretation of metabolic and biomechanical results. In general, the present work demonstrates the potential of lightweight passive exoskeletons to make overhead work activities easier for workers.

### 4.1. Metabolic Parameters

For the two investigated tasks, T1 and T2, the physiological parameters O_2_ rate and heart rate served as indicators of metabolic energy consumption. Due to the moderate working intensity and durations not exceeding 30 min, a nearly constant aerobic metabolism can be assumed [25,26]. Furthermore, similar metabolic rates did not vary significantly, permitting a direct comparison of values for the WE and WOE conditions.

At the global level, the measured O_2_ and heart rates demonstrated that the use of the exoskeleton led to a significant reduction in metabolic effort during the specific physical activity performed in task T1 and T2. Thus, it may be concluded that the investigated physical activities are less fatiguing overall when using the studied exoskeleton. So far, only one study compared metabolic parameters for overhead work activities with and without an exoskeleton. Since Maurice et al. [12] used the same exoskeleton, it even allows for a direct comparison. They observed the same trend but with an even larger reduction in O_2_ consumption (about 33% vs. 11%) and heart rate (about 19% vs. 5%) which might be due to the different designs of the experiments. 

### 4.2. Biomechanical Parameters

The findings regarding the activation of the ten shoulder girdle muscles analyzed allow for a complex evaluation of both the usable shoulder joint range of motion and the neuromuscular effort required to perform static and semi-static overhead work activities with an exoskeleton.

In general, the kinematics of the involved joints should be considered when interpreting the muscle activation patterns. The found joint angles were nearly identical for shoulder anteversion for T1 and T2 and elbow flexion for T1 for WE and WOE conditions. In conjunction with moderate differences in shoulder adduction angles (T1 and T2) and elbow flexion (T2), this indicates that the lengths of the analyzed muscles are almost the same in both test conditions. The force generated by a skeletal muscle basically depends on (1) muscle length (moderate differences between WE and WOE), (2) muscle contraction velocity (approximately zero for static and semi-static tasks) and (3) muscle fiber activation. Our findings allow for the plausible conclusion that the drastic reduction in EMG amplitudes in WE correlated with fewer activated muscle fibers and, thus, presumably less muscle forces generated [27]. This implies that reduced muscle forces were required to perform the tested overhead work activities with the exoskeleton. 

The most drastic reduction of the EMG amplitudes was seen in the deltoid and biceps brachii muscles, which are the most important generators of the forces required to perform shoulder anteversion [28]. The same functional role can be assigned to the deltoid and serratus muscles for shoulder abduction that also exhibited large EMG amplitude reductions of about 40%. In addition, the significant reduction in EMG amplitudes of the other muscles (trapezius, latissimus dorsi, external obliquus muscles) indicated that the use of the exoskeleton also led to a reduction in the necessary muscle activation to stabilize the shoulder girdle and upper trunk region. In general, the results of the EMG analysis are comparable with the reductions in muscle activation reported for other exoskeletons. For example, Engelhoven et al. [8] reported reductions between 18 and 80% (depending on support level and muscle; deltoid and trapezius muscles), Otten et al. [11] found reductions between 27 and 58% (deltoid muscle) and Kim et al. [7] between 24 and 49% (deltoid and trapezius muscles). Another lab study with PAEXO investigated also the lower erector spinae muscle but did not find a significant change in muscle activation when using the exoskeleton [12]. Therefore, it can be assumed that only slight potential adverse effects may have to be expected with the use of PAEXO. One of the reasons may be found in the low weight of the decvice. 

From the orthopedic point of view, the reduction in the EMG amplitude and, thus, presumably the force generated by the deltoid muscle appears to be clinically meaningful. First, decreased muscle forces lead to a reduction in the resultant joint compression force, in this case on the glenohumeral joint, which can be considered an unloading effect [29]. A further, more specific effect applies to the association of long-term or frequent overhead activities and the development of joint impairment such as impingement syndrome and rotator cuff injuries. The overhead elevation movement of the upper arm, a combination of anteversion and abduction, is mainly generated by the force of the deltoid muscle [28]. At the same time, while elevating the arm, the deltoid muscle induces a vertical dislocating force to the humeral head. This force is usually neutralized by the centering force of the rotator cuff. The vertical dislocation force acting on the humeral head is an important factor in the pathogenesis of subacromial pathology [30]. Therefore, reducing the dislocating force of the deltoid muscle by using the exoskeleton may have preventive effects on the development and treatment effects on existing conditions like impingement syndrome and rotator cuff injury.

An interesting interconnection between the EMG and metabolic effects is represented by the Muscle Fatigue Index (MFI) that assesses symptoms of fatigue over time during static tasks. It has low sensitivity and, therefore, is rarely used in studies of muscle function [23]. However, the differences in MFI showed a significant reduction in muscle fatigue during overhead tasks when the exoskeleton is used. Again, the effects were most pronounced for the deltoid and biceps brachii muscles. This also indicates that these muscles are the most significant ones for holding the upper arm in the position required to execute overhead work activities in T1.

### 4.3. Limitations

The fact that the tests were conducted with a group of healthy young subjects in two specific overhead work activities can be seen as a general limitation of the study. Further studies should be conducted to address the question whether these results can be generalized to subjects of older age groups and for other overhead activities [14,15]. Furthermore, previous studies demonstrated that decreased loads in one part of the body may result in increased strain to other areas due to weight transfer by the exoskeleton and its own weight [27,31]. This question was already studied for the lightweight exoskeleton investigated in the present study, finding no adverse effects [12]. In addition, standardized evaluation methods are still lacking due to the novelty of exoskeletons in the working environment [12]. Direct comparisons to other studies [7,8,9,11,12,31] are therefore only possible to a limited extent.

## 5. Conclusions

This study demonstrated the biomechanical and metabolic benefits of an exoskeleton designed to support overhead work activities. Based on the results, improved physiological conditions and an unloading effect on the shoulder joint and muscles may be assumed. An aspect worth noting is the fact that the motion patterns are similar when comparing the overhead activities with and without the device. This indicates that the exoskeleton does not impose any unnatural or unwanted motion patterns or restrictions on the user. 

The results and the significantly lower weight of the exoskeleton are promising indicators that the exoskeleton may be a good solution to reduce shoulder WMSD among workers who have to perform overhead tasks on a regular basis.
